# Accelerating AI for science: open data science for science

**DOI:** 10.1098/rsos.231130

**Published:** 2024-08-21

**Authors:** Neil D. Lawrence, Jessica Montgomery

**Affiliations:** ^1^ Department of Computer Science and Technology, University of Cambridge, Cambridge, UK

**Keywords:** AI, open science, AI policy, AI for science

## Abstract

Aspirations for artificial intelligence (AI) as a catalyst for scientific discovery are growing. High-profile successes deploying AI in domains such as protein folding have highlighted AI’s potential to unlock new frontiers of scientific knowledge. However, the pathway from AI innovation to deployment in research is not linear. Those seeking to drive a new wave of scientific progress through the application of AI require a diffusion engine that can enhance AI adoption across disciplines. Lessons from previous waves of technology change, experiences of deploying AI in real-world contexts and an emerging research agenda from the AI for science community suggest a framework for accelerating AI adoption. This framework requires action to build supply chains of ideas between disciplines; rapidly transfer technological capabilities through open research; create AI tools that empower researchers; and embed effective data stewardship. Together, these interventions can cultivate an environment of open data science that deliver the benefits of AI across the sciences.

## 1. Introduction

The information revolution has fostered a wave of progress in artificial intelligence (AI), driven by the ability to collect, store, exchange and interconnect different datasets. While the mechanization of the industrial revolution required coal and heat engines, informational mechanization deploys data and data engines to generate actionable knowledge. This process requires a combination of mathematical and computational modelling, and a combination of skillsets that falls across traditional academic boundaries.

Access to data, development of increasingly powerful computer systems, and algorithmic advances have contributed to rapid progress in AI over the last 10 years. The term ‘AI’ today describes a cluster of different methods and tools. Much of the recent progress in AI has been driven by advances in machine learning, an approach to AI focused on training computer systems to perform complex tasks by learning from data. In public and policy debates, these terms are used interchangeably. This paper uses both to describe the use of AI for scientific discovery, acknowledging that data-driven methods are a primary focus for many of today’s AI for science efforts.

Data have long been at the heart of the scientific method. Science advances understandings of the world by collecting, analysing and interrogating data. Science could, therefore, be expected to be at the vanguard of the information revolution, taking advantage of new sources of data to extract new insights about the world around us. Many of today’s pressing research and policy challenges demand the ability to analyse, understand and identify levers that influence complex systems. For researchers across domains, AI-enabled analytical tools open the possibility of generating novel insights into the dynamics of such systems, leveraging data to drive scientific progress. For decision-makers in research, policy and industry, the use of AI to accelerate discovery offers a mechanism to boost scientific productivity and generate innovative solutions to issues of scientific and societal concern.^1^ The EU’s Innovation Missions, for example, set out crucial research-policy challenges in climate adaptation, cancer prevention and treatment, ocean protection, smart cities and soil health, where innovative solutions are needed to sustain human health and wellbeing. AI could supercharge research and development activities across these areas, by offering advanced analytical techniques or decision-support systems.^2^

Rallying around these ambitious goals to generate new understandings of complex physical, biological, environmental, social and technological systems, across disciplines researchers are making use of AI technologies to interrogate new data sources [1,2]. These efforts have yielded high-profile successes that suggest the significant potential of AI for science. The AlphaFold project, for example, leveraged AI methods to make impressive progress in predicting the three-dimensional structure of proteins from their amino acid components [3]. A growing pool of projects illustrate the breadth of potential applications in AI for discovery, from archaeological to zoological research.^3^ However, achieving high levels of adoption across research disciplines remains a distant goal.

In some respects, this pattern is unsurprising. The translation of innovation to adoption is neither a simple nor linear process. Lessons from the history of technological change [4] and analyses of the last 10–15 years of economic growth [5] highlight the complexity of the processes underpinning technological diffusion, and the web of relationships between innovation, practice and productivity that influences its success.

The question that follows is how to create the conditions that enable the diffusion of AI across the sciences. This requires consideration of the environment into which AI is being deployed. To explore this environment, this paper draws together perspectives from previous waves of technology adoption, AI and policy research to consider what infrastructure can enhance the diffusion of AI across the sciences. It suggests a framework for enabling the adoption of AI for science. The intention here is not to present a detailed adoption roadmap, but to articulate an approach to AI in science—rooted in open data science—that can build capabilities in this field over the long term.

## 2. Understanding the AI productivity puzzle

In 1987, the economist Robert Solow observed that ‘You can see the computer age everywhere but in the productivity statistics’ [6]. Solow’s productivity paradox described the disconnect between the pace of technological innovation arising from the computing revolution and the apparent stagnation of the US economy. This pattern continued to the 1990s, until widespread adoption of information technologies began to transform traditional business processes, such as supply chain and distribution [7].

Similar patterns can be seen throughout the history of innovation. There is a lag between invention and widespread benefit, as people and organizations reorganize around new technologies, finding new processes and ways of working. While innovation brings productivity benefits, these benefits depend on patterns of adoption and can take decades to emerge.^4^ The process of reorganization—who adapts in what ways—also influences the extent to which the benefits of innovation are shared across sectors and societies.^5^

In science, it is already possible to see varied patterns of AI adoption across disciplines. Large-scale modelling and data challenges can be found at the core of domains such as astronomy (e.g. [8]), and climate science,^6^ while computational biology has a well-established culture of data science for scientific discovery, with large-scale projects such as the Human Genome Project helping to embed a culture of using data science for science. Today, projects such as AlphaFold [9] extend the frontiers of these efforts, demonstrating how AI can be applied to tackle long-standing scientific challenges. These successes act as a beacon, inspiring researchers with the possibilities of AI for scientific discovery. Translating this success into wider scientific progress will require further work to embed AI in those domains without such a strong tradition of deploying data science methods.

In some regards, this disciplinary dynamic—early adopter domains reaping the benefits of new technologies while others have yet to engage—mirrors well-established patterns of technology diffusion in other sectors.^7^ When considering how to promote the diffusion of innovation across industry sectors and organizations, policymakers have looked to stimulate both supply and demand,^8^ through strategies that include leveraging supply chains as a pathway for spreading innovation; enhancing technology transfer through university–business collaboration; and building human capital by spreading skills across companies [10].

While dealing with different market dynamics and policy frameworks, these analyses offer a lens for those promoting AI as a tool for enhancing scientific productivity, helping to identify relevant institutional, technical or policy levers for change. The results of these efforts suggest that: (i) to achieve a step-change in scientific discovery using AI, adoption across domains will be necessary, and interventions must embrace both early-adopter disciplines and the long tail; (ii) stimulating demand is essential, through supply chains of ideas and institutional interventions that cultivate a desire to use AI for science; and (iii) further work is needed to enhance the absorptive capacity of disciplines to make use of AI, through efforts to build skills and human capital. These lessons can help provide a framework for supporting the adoption of AI for scientific discovery. Before designing such frameworks, however, researchers wishing to deploy AI for science must consider whether their AI tools are fit for purpose.

## 3. Deploying AI in science

Today’s AI methods can deliver impressive outcomes when trained to perform defined tasks in controlled environments. Automating more sophisticated tasks typically requires combinations of machine learning sub-components, creating complex interactions between data, algorithms, models and system outputs. This complexity contributes to a gap between user aspirations for the tasks that AI might perform and the safety and reliability of AI systems in deployment.

This disconnect has already resulted a range of AI failures in real-world contexts. Failure modes vary, arising at each stage of the AI development pipeline, from understanding user needs, to managing data quality, to maintaining performance levels in changeable environments or anticipating user interactions [11]. These failures can have significant implications—for individuals that might be subject to physical harm, for communities that might suffer discrimination or marginalization, for organizations reliant on AI for business processes and for society as a whole, if AI contributes to wider social disruption.^9^

The use of AI in research and development efforts connected to COVID-19 response highlights the challenges of designing and implementing AI systems that can perform well in real-world contexts. In the UK, AI played little—if any—role in the response to COVID-19 [12–14]. Where systems were created with the intention of improving healthcare outcomes, problems with data quality, methodological issues in the design of AI models and deficiencies in reporting practices all contributed to the development of a suite of AI systems that were generally unfit for use in clinical settings [14–16]. Researchers working in other disciplines report similar issues, highlighting the limited usefulness of some existing training datasets for research challenges, the potential for AI to reinforce inaccuracies or bias in data and the vulnerability of some existing AI methods to adversarial attacks or other issues with robustness [17]. For AI to be successfully deployed in research, AI for science needs policies, practices and methods to tackle these issues. A framework for deploying AI in science that acknowledges these real-world deployment challenges and provides mechanisms to build capability—both in the application of existing AI tools and the development of next-generation AI tools—can help increase the effectiveness of AI for science projects.

The need to overcome the limitations of today’s systems and practices also offers an opportunity to envisage a new wave of progress in AI’s technical capabilities, creating advanced analytical tools that can be deployed in the service of scientific discovery. This research agenda in AI for science spans [18]:

— *Building the technical foundations of AI for science*. The central goal of AI for science is to leverage insights from data to generate new scientific knowledge. Generating this knowledge requires technical developments to increase the analytical power of today’s AI for science tools. Areas for progress include: advances in simulation and emulation to allow researchers to interrogate the workings of complex systems; causal AI methods that can detect scientifically meaningful structure in data, analysing not only what patterns exist but also why they emerge; and the ability to formalize concepts such as interpretability or uncertainty quantification [19].
— *Interfacing with domain knowledge*. Translating insights from data to scientifically actionable information requires mechanisms for information exchange between researcher and AI. In pursuit of this goal, there are already model design strategies that help encode domain knowledge in AI systems, for example making use of known physical laws or invariances. More sophisticated techniques are needed to access and leverage the tacit knowledge that researchers also bring to their work. Additional insight can be gained by integration of simulations, for example mechanistic models or counterfactual simulations either within the model’s inductive bias or through data directly generated from these systems. A combination of new learning strategies, system designs and user interfaces open the possibility of creating analytical assistants with a form of ‘theory of mind’, able to identify a researcher’s goals or interests, even when these might be unspoken or uncertain [20].
— *Enabling adoption*. Widespread adoption of generalizable AI tools will require both the technical progress set out above and mechanisms to facilitate their access and use. Libraries, toolkits and user guides play an important role in capturing the knowledge generated by the AI for science community and supporting researchers to overcome the practical challenges of deploying AI.

Such advances offer the possibility of both driving forward the science of AI and creating AI tools that can better serve the needs of researchers and organizations deploying AI.

## 4. Creating an infrastructure for diffusion

Accelerating the adoption of such next-generation AI for science tools requires an engine for diffusing these innovations across the sciences. Open data science for science offers a framework to deliver this diffusion, based on five pillars:

— supply chains of ideas to advance AI for science methods and applications;
— transfer of technological capabilities from methods to application communities through open toolkits;
— capability building that empowers researchers to deploy AI for their science;
— data-first culture that delivers effective data stewardship; and
— interfaces between users and AI.

### 4.1. Supply chains of ideas to advance methods and applications

Connections between disciplines are central to the success of AI in science. Supply chains of ideas are necessary to take innovative ideas from their source to where they can be successful adopted [21]. Sustained engagement between disciplines plays an important role in building these supply chains, by increasing mutual understanding of what different disciplines can deliver. Central to their success is that ideas can connect in different directions: that innovative AI methods can be deployed in areas of scientific need, and that scientific needs can be used to inspire innovations in AI. The result should be a dynamic interdisciplinary community where advances in AI support advances in science, and vice versa, fuelled by collaborations between domain and AI experts that deliver benefits to both.

Multi-disciplinary work also brings challenges, many of which are well-characterized in studies of research culture and policy. In the context of AI adoption, a particular challenge is the different languages employed by different domains for related technical ideas. The use of jargon in specific fields and assumptions around what is canonical knowledge—versus what specifics might need explaining—act as barriers to collaboration.

Data offer an opportunity to overcome these barriers by providing a focal point for convening different disciplines. Even where data do not exist, the process of exploring what data might be required to answer a question can provide a shared point of reference for scientists approaching a research area from different disciplinary backgrounds [22]. Spaces for such conversations and collaborations are necessary to create an environment in which multi-disciplinary collaborations can emerge, supported by institutional research cultures that recognize and reward individuals working at the interface of different domains.

The result of overcoming these barriers is research at the interface of AI and the sciences that pushes the boundaries of AI capabilities and disciplinary knowledge. Examples include new reflections on the nature of biological understanding in the context of AI progress [23], advances in AI methods to enable their application for research^10^ or ideas for future areas of inquiry [24].

### 4.2. Transfer of technological capabilities through investing in tools and toolkits

In environments that do not naturally encourage such multi-disciplinarity, machine learning can become intellectually isolated from the sciences in which it is deployed. Those working on machine learning techniques within a specific scientific domain are often separated from the wider machine learning community, lacking access to the expertise they need to avoid reinventing the wheel or chasing phantoms in their efforts to deploy useful machine learning methods.

To help correct this dynamic, further efforts are needed to make new analysis methodologies available as widely and as rapidly as possible. Those creating new AI techniques must also ensure they can be operated safely and reliably in deployment, employing methods and design practices that increase the robustness of the toolkits they produce. This requires an institutional environment that supports publication of new methods with few constrictions on their use and with relevant explanatory material. Team science can play a role in addressing these concerns, bringing together a mix of expertise in AI, science and engineering to create accessible toolkits in AI for science.

Kuhn’s analysis of the structure of scientific revolutions suggests that scientific paradigms are stored in books, but that modern information infrastructure has caused a shift towards the storage of scientific knowledge in software (in the form of models) or data [25,26]. Computational biology is one domain that has led in provision of these data and models derived from it. One example of such an approach can be seen in the Structural Antibody Database (SAbDab),^11^ driven by the work of the Oxford Protein Informatics Group, which maintains data sources as well as building machine learning models from them [27]. Kuhn associated the process of ‘normal science’ as solving within a paradigm, historically defined by textbook knowledge [25]. Major scientific projects such as AlphaFold are also shifting the paradigm of science itself. While headline science is often conducted in these one-off projects, many scientists continue to pursue the puzzles that are defined by these works. It is the shifting nature of the paradigm and its representation in software and data that has effects well beyond these larger well-known achievements.

### 4.3. Capability building that empowers researchers to use AI

While further progress in AI methods is necessary, for many scientists access to AI is restricted not by the lack of availability of better AI tools, but by the technical inaccessibility of existing methods. A fundamental challenge for the field is bridging this gap between the data analyst and the scientist. New approaches are needed to equip scientists with the fundamental concepts that will allow them to explore their own areas of research using a complete mathematical and computational toolbox. Training this cohort of AI practitioners, who are empowered to deploy AI tools for their research through research-focused teaching and learning activities, will require teaching methods that fall outside the scope of business-as-usual university training. For example, from 2020 to 2023 the Accelerate Programme for Scientific Discovery trained over 400 researchers in data science and AI. This training offer has included:

— taught courses on methods in data science and machine learning;
— practical training in how to build data pipelines, package and publish software and hands-on sessions in how to use Large Language Models for research; and
— advice and mentoring in the practical application of data science and machine learning in science.^12^

Recent advances in generative AI methods, such as Large Language Models, are also likely to disrupt this landscape as they provide new interfaces between humans and data that provide opportunities for better data representation. This also comes with risks of misrepresentation, discussed further below.

### 4.4. Data-first culture

The core of the information revolution is the ability to monitor, store, interconnect and analyse large interacting datasets. The use of many of today’s most prominent AI methods in science will rely on access to well-curated and interconnected data sources. Policies for research data management are now well-established in research institutions. While its merits might not be universally accepted by individual scientists, funding agencies today encourage widespread data sharing.^13^ Aspirations for wider deployment of AI for science underscore the importance of effective data governance, with good data management practices requiring further uptake across disciplines.

Many of these existing frameworks for data governance focus on the management of ‘traditional’ data sources—data collected for research with a specific purpose in mind. As the variety and volume of data with potential application in research grows, institutions and researchers must also grapple with how to steward the use of new data sources. Individuals and organizations today generate data from a range of daily activities, and there are opportunities to use so-called happenstance data in research. With such data not having been actively collected with a research question in mind, extra care is needed in their analysis, to prevent misleading results.^14^ Use of happenstance data can also generate new ethical concerns, if its integration and analysis yields sensitive insights about individuals or creates other concerns around privacy.^15^

These changing opportunities and challenges in relation to data use highlight some of the fractures in the current data governance landscape. There are open questions about:

— what further policy interventions can promote data accessibility while ensuring its trustworthy governance;^16^
— what incentives can help promote adoption of existing interventions, such as the FAIR principles,^17^ that aim to support data sharing and use; and
— what research practices can help ensure the responsible deployment of AI in science, in the context of today’s needs for careful data stewardship.^18^

In response to concerns about governance of potentially sensitive data and the range of operational barriers to data access that can arise across organizations, synthetic data have attracted interest as a potential alternative data source. These artificially generated data are designed to mimic the characteristics of a real-world dataset, with the aim of providing a data resource that can help develop machine learning algorithms [28]. The hope for such data is that their use would offer a route to addressing some of the ethical concerns associated with personal or commercially sensitive data, such as maintaining privacy or tackling bias, enabling faster progress in the development of machine learning systems [29]. In areas such as healthcare, for example, such data could be used to simulate the impact of different policy interventions on health outcomes [30]. However, alongside these hopes for synthetic data, recent years have brought growing understanding of the limitations of these resources, both in terms of their ability to address concerns around privacy and representativeness of real-world datasets.^19^ While a useful tool for machine learning development in some contexts, synthetic data will not circumvent the need for trustworthy data governance practices.

New data stewardship mechanisms will be necessary to assimilate complex information resources while managing them in line with legal and ethical obligations [31]. Institutional innovations, such as data trusts, offer a route to better aligning public expectations in relation to data governance with its proposed uses [32] and pilot projects are already trialling these approaches to research data governance.^20^ In the long term, such data intermediaries offer a mechanism to address both the demand for access to data and the need to align data access arrangements with public interests and expectations. While these mechanisms develop, organizations can help foster a data-first culture through incentives for trustworthy, open data stewardship and clear practices for delivering such stewardship.

### 4.5. Interfaces between users and AI

In science, the interface between data and human has always been subject to potential misrepresentation. Mark Twain attributed the quote ‘There are three types of lies: lies, damned lies and statistics’ to Benjamin Disraeli, but in practice, this sentiment can be found in several different forms across the late nineteenth century. It reflects the manner in which the ‘science of state’ could be corrupted by numbers that give humans a non-representative impression of the underlying challenges. The modern equivalent of this quote would be ‘lies, damned lies and big data’, as the challenges of misrepresentation have shifted with both the quantity of data that can be collected and the use of computer-driven interpolations that can incorporate new sources of bias in their models.

This challenge leads to a ‘big data paradox’ where increasing data collection results in less understanding, as the scale of data available is beyond an individual human’s ability to assimilate, and yet the data may still misrepresent the underlying phenomena. Similarly, large models lead to a ‘big model paradox’ where more and more aspects of the underlying phenomena are encoded in computer models, but the complexity of the model moves beyond an individual human’s understanding. This phenomenon is related to a challenge that, in the context of computer systems, Jonathan Zittrain refers to as intellectual debt [33]. The main message is that larger is not necessarily better when greater size moves models beyond our traditional (often statistical) methods of verification.

Generative AI models offer the potential to both make this problem worse or improve the challenge significantly depending on how they are deployed. Their capabilities to wield language promise a future where the relevant information about a dataset or a model challenge could be extracted in the same way that humans exchange information with each other, i.e. through conversation. If successfully deployed, such models could enhance researchers’ ability to interact with AI systems, to interrogate their outputs and to explore the implications of those outputs.

However, generative AI also opens a new front for the possibilities of misrepresentation, with associated challenges of understanding how humans exchange information and uncertainties through this medium. The tendency of generative models to provide convincing ‘hallucinations’ as outputs calls into question their accuracy and reliability, with implications for how they can be deployed responsibly in the scientific context [34]. Concerns about bias [35], privacy and security [36] also influence how generative AI systems can be adopted responsibly for research [37].

## 5. Conclusion

Twelve years ago, the Royal Society’s report *Science as an open enterprise* set an agenda for embedding the principles of open science in a changing scientific environment. Its calls for more recognition for the value of data management, standards for information sharing and new software tools, among other areas for action, sought to translate excitement about the potential of big data to a new revolution in open science [38]. The decade since its publication has seen both significant progress in the volume of data available to researchers and the technical capabilities of AI as a tool to analyse it. It has also highlighted the fault lines in research and innovation policy—in research culture, funding and incentivization, data management and open science—that continue to affect the adoption of data science across research disciplines. If not addressed, these will hold back the potential of AI in science. Over the same period, concerns about the ‘reproducibility crisis’ in research have continued to emerge in different fields [39], including AI for science [40]. In this wider context, open science is a crucial tool to maintain scientific rigour, by enabling researchers to build on—or challenge—research outputs and evaluate the reliability of AI methods before deployment.

There is no ‘silver bullet’ for the challenges of deploying AI for scientific discovery. However, the interventions described above point to an approach that—when combined with the appropriate domain expertise—can help address these issues in the long term through new communities of research and practice. This approach is *open data science* [41]].

The open-source community has played a central role in enabling today’s technological environment. Microsoft’s quasi-monopoly on desktop computing was disrupted by open source software that would have been unfeasible for any single organization to create; it has been estimated that the development cost of a full Linux system would be $10.8 billion dollars [42]. Regardless of the veracity of this figure, it is clear that Linux—and other open-source software—has been an important enabler of innovation, by providing a foundation on which Apple, Google and others could build.^21^ In the modern Internet, tools such as GitHub, Jupyter notebooks, preprint repositories such as arXiv and bulletin boards such as Reddit continue this tradition of seeking routes for early distribution and comment on material.

Open data science aims to bring the same spirit of community resource generation and assimilation to capitalize on the underlying social driver of this phenomenon: many talented people would like to see their ideas and work being applied for the widest benefit.

AI researchers and data scientists can help bring about an environment of open data science through widespread distribution of ideas under flexible BSD-like licenses that give scientific partners as much flexibility as possible to adapt methods to their own circumstances, and widespread distribution of teaching materials. Domain experts play a role in seeking opportunities to pick up these methods, engaging with new approaches to professional development and investing in disciplinary data curation efforts.

Institutions can provide incentive structures that reward researchers for experimentation with the use of AI, providing career pathways for those pursuing this deeply interdisciplinary work, creating spaces for those working in AI and those working in scientific domains to exchange knowledge and ideas, and investing in education programmes that address the gaps in current expertise.

Open data science should be an inclusive movement that operates across traditional boundaries between academic disciplines, and between companies and academia. It could bridge the gap between ‘data science’ and science, and address the barriers to large-scale analysis of data in areas of pressing social need (climate; health), spurring a new wave of innovation in both the public and private sector.

## Data Availability

This article has no additional data.

## Footnotes

^1^
These aspirations come amidst an active debate about whether scientific productivity is declining. See, for example: Boeing P, Hünermund PA. 2020 global decline in research productivity? Evidence from China and Germany. *Econ. Lett*. **197**, 109646. https://doi.org/10.1016/j.econlet.2020.109646 and Oxford Economics. 2021 *The State of Scientific Research Productivity: How to Sustain a Critical Engine of Human Progress*. See https://www.oxfordeconomics.com/resource/the-state-of-scientific-research-productivity/.

^2^
For an overview of the associated application areas, see: ELISE Consortium. 2023 *AI for European Grand Challenges*. See https://www.elise-ai.eu/sra-refresh/ai-for-european-grand-challenges.

^3^
Examples of current projects from across disciplines are available on the blog for the Accelerate Programme for Scientific Discovery, here: https://acceleratescience.github.io/blog.

^4^
For a discussion of the economic effects that may influence the impact of AI-enabled automation on work, see: Acemoglu D, Restrepo P. 2018 A*rtificial Intelligence, Automation and Work (NBER Working Paper No. 24196)*. Cambridge, MA: National Bureau of Economic Research. On similar themes, Brynjolfsson *et al*. consider the impact of lags in implementation of AI on productivity growth today: Brynjolfsson E, Rock, D, Syverson C. 2017 *Artificial Intelligence and the Modern Productivity Paradox: A Clash of Expectations and Statistics (NBER Working Paper No. 24001)*. Cambridge, MA: National Bureau of Economic Research. These are summarized in: The Royal Society. 2018 The impact of AI on work: implications for individuals, communities, and societies. See https://royalsociety.org/news/2018/09/the-impact-of-AI-on-work/.

^5^
Crafts explores the lag between technological inventions that were significant in the British Industrial Revolution and subsequent productivity gains, considering the role of labour and energy costs in contributing to this dynamic. Crafts, N. 2010 The Contribution of New Technology to Economic Growth: Lessons from Economic History. *CAGE Online Working Paper Series 01, Competitive Advantage in the Global Economy*. See https://warwick.ac.uk/fac/soc/economics/research/centres/cage/manage/publications/01.2010_crafts.pdf. Mokyr *et al*. also consider the interlinkage of social, economic and cultural changes in the context of the impact of technology on working life. Mokyr J, Vickers C, Ziebarthm NL. 2015 The history of technological anxiety and the future of economic growth: is this time different? *J. Econ. Perspect*. **29**, 31–50. (doi: 10.1257/jep.29.3.31). Harford considers how these dynamics influenced the use of electricity in manufacturing. Harford, T. 2017 *Why didn't electricity immediately change manufacturing*? See https://www.bbc.co.uk/news/business-40673694 (accessed 11 April 2024).

^6^
See, for example, work of the Met Office Informatics Lab: https://www.informaticslab.co.uk.

^7^
As Andy Haldane, then Chief Economist at the Bank of England, described in 2018 in relation to innovation ‘In the fullness of time, innovation should be expected to diffuse through the economy, lifting all boats. That has been the lesson of every industrial revolution. Yet in the UK, this technological trickle-down, from frontier to tail, appears to have dried up. A lengthening flotilla of boats has remained in dry dock. The diffusion engine appears, for them, to have seized up.’ Haldane, A. *The UK’s Productivity Problem* [10]; available at: https://www.bankofengland.co.uk/-/media/boe/files/speech/2018/the-uks-productivity-problem-hub-no-spokes-speech-by-andy-haldane.

^8^
A recent analysis of this challenge by the Institute for Government characterized the necessary response as follows: ‘Productivity gains need to find their way into companies throughout the economy, diffused through market forces and motivated management seeking better ways to service growing demand.’ Institute for Government. 2021 *Productivity: firing on all cylinders*. See https://www.instituteforgovernment.org.uk/sites/default/files/publications/productivity-restoring-growth.pdf.

^9^
To explore incidents of AI failure, see *Partnership on AI: AI incident database*, at https://incidentdatabase.ai.

^10^
For example, in environmental science, as introduced by Hickman S. here: https://acceleratescience.github.io/2023/02/27/using-ai-to-aid-causal-methods-in-environmental-science.html.

^11^
See: https://opig.stats.ox.ac.uk/webapps/sabdab-sabpred/sabdab/about.

^12^
For further details, see: https://acceleratescience.github.io/resources.html.

^13^
For example, UKRI ‘expects research data arising from its funding to be made as open as possible and as restricted as necessary’ and sets out a range of principles and policies in support of this aim: https://www.ukri.org/manage-your-award/publishing-your-research-findings/making-your-research-data-open/.

^14^
For example, if we assume the politics of active users of Twitter is reflective of the wider population’s politics, then we may be misled.

^15^
Some issues in the governance of happenstance data are explored in The DELVE Initiative, *Data Readiness: Lessons from an Emergency*. 2020; DELVE Report No. 7. Published 24 November 2020. See https://rs-delve.github.io/reports/2020/11/24/data-readiness-lessons-from-an-emergency.html.

^16^
For example, the UK Government set out an ambition to unlock the value of data through enhanced data sharing in the National Data Strategy. 2020 UK Government, *National Data Strategy*. See https://www.gov.uk/government/publications/uk-national-data-strategy/national-data-strategy#data-2-1 (accessed 11 April 2024).

^17^
Available at: https://www.go-fair.org/fair-principles/ (accessed 11 April 2024).

^18^
For example: Bano M, *et al.* 2023 Investigating responsible AI for scientific research: an empirical study. *arXiv preprint*. https://doi.org/10.48550/arXiv.2312.09561.

^19^
For example, Jordon *et al* summarize how synthetic data do not guarantee privacy and may distort the information represented in real-world datasets. Jordon J. *et al*. 2020 Synthetic data – why, why, and how? Report commissioned by the Royal Society. See https://royalsociety.org/-/media/policy/projects/privacy-enhancing-technologies/Synthetic_Data_Survey-24.pdf (accessed 11 April 2024).

^20^
See, for example, the Born in Scotland Data Trust: https://warwick.ac.uk/fac/soc/law/research/projects/scotland-data-trust/ (accessed 11 April 2024).

^21^
Android is based on Linux; OSX is based on FreeBSD.
